# Identification and Quality Assessment of Chrysanthemum Buds by CE Fingerprinting

**DOI:** 10.1155/2015/517402

**Published:** 2015-04-30

**Authors:** Xiaoping Xing, Dan Li

**Affiliations:** Department of Chemical and Biological Engineering College, Yancheng Institute of Technology, Yancheng 224051, China

## Abstract

A simple and efficient fingerprinting method for chrysanthemum buds was developed with the aim of establishing a quality control protocol based on biochemical makeup. Chrysanthemum bud samples were successively extracted by water and alcohol. The fingerprints of the chrysanthemum buds samples were obtained using capillary electrophoresis and electrochemical detection (CE-ED) employing copper and carbon working electrodes to capture all of the chemical information. 10 batches of chrysanthemum buds were collected from different regions and various factories to establish the baseline fingerprint. The experimental data of 10 batches electropherogram buds by CE were analyzed by correlation coefficient and the included angle cosine methods. A standard chrysanthemum bud fingerprint including 24 common peaks was established, 12 from each electrode, which was successfully applied to identify and distinguish between chrysanthemum buds from 2 other chrysanthemum species. These results demonstrate that fingerprint analysis can be used as an important criterion for chrysanthemum buds quality control.

## 1. Introduction

Chrysanthemums, colloquially known as mums, are herbaceous perennial flowering plants and have been cultivated for over 3 millennia. Chrysanthemums include more than 3000 varieties [1], including* Ammobium alatum*, perennial chamomile,* Aster novi-belgii*, and* Calendula officinalis*, which come from different regions, flower in different seasons, and may contain different active compounds. Chrysanthemum buds are one of the highest grades of chrysanthemum in use. Chrysanthemum buds are an important component in many traditional Chinese medicine (TCM) formulas [2] for its therapeutic effects, which include antioxidant, anti-inflammatory, antiviral (including HIV), antimutagenic, anticarcinogenic, antihepatotoxic, and antiaging activities [3]. Chrysanthemum buds are also a common health food/supplement used by many consumers [4] for “scattering cold,” “cleaning heat and toxin,” and “brightening eyes,” which are considered beneficial to human health.

Significant amounts of biologically active compounds have been found in chrysanthemum buds that play important roles in human body, mainly including flavonoids, carbohydrate, and essential oils [5]. Among these compounds, chlorogenic acid, luteolin, and glucoside have been confirmed to possess a variety of biological activities [6]. Traditionally, these active components were used to evaluate the quality of raw plant material. However, owing to the fact that there are hundreds of complex active components in chrysanthemum buds, it has been extremely difficult to identify all these substances and carry out quantitative analyses on them individually. What is more, the chemical composition of chrysanthemum buds can differ between different varieties. As a result, it became necessary to develop a new technology to capture the total chemical composition of chrysanthemum buds and to identify chrysanthemum varieties and verify their authenticity.

Fingerprinting is a method to capture total chemical information of herbs by chemical analytical techniques and is displayed as spectrograms, electropherograms, and other graphs. Fingerprint analyses produce a representative “fingerprint” that contains the greatest amount of information possible to accurately represent a sample and distinguish it from others. Fingerprint analysis of medicinal herbs has been the optimal measurement for identifying and assessing the variety and quality of the plants. Fingerprint analysis has been accepted as a strategy for the assessment of herbal medicines for the evaluation of medicinal products for herbal preparations by the U.S. Food and Drug Administration (FDA) [7] and the European Medicines Agency [8]. In China, the former State Drug Administration (SDA) also began to develop fingerprints of raw materials as a standard of quality control in 2000 [9].

Recently, several techniques have been developed which can characterize the nature and chemical composition of substances. HPLC [10] and GC [11], prime techniques used for fingerprint analysis, have high precision, sensitivity, and reproducibility. However, sample preparations, including preconcentration and derivatization, are often time-consuming, complicated, and troublesome. Thin layer chromatography (TLC) [12] is a commonly used technique for screening of herbal liquid extracts. The ultra-performance liquid chromatography (UPLC) [13] approach has some advantages over HPLC, GC, and TLC, including a large decrease in analysis time and solvent consumption, the possibility of obtaining high efficiencies, and the ability to resolve coeluting compounds. However, its drawbacks include increased back-pressure and the availability of only few stable stationary phases.

CE technology has been widely applied to the characterization of diverse samples due to its low cost, minimal sample volume requirement, short analysis time, and high separation efficiency [14–16]. Electrochemical detection (ED) is a commonly used chemical detection method because of the small size of both the detector and control instrumentation and low power demands [17]. CE coupled to electrochemical detection (CE-ED) is a useful technology offering high sensitivity and good selectivity for electroactive analytes. Based on the two main kinds of active compounds in chrysanthemum buds, flavonoids and polysaccharides including the hydroxyl (–OH) groups are electroactive at carbon and copper electrodes, respectively, which suggests that CE-ED is an appropriate method to investigate the chemical fingerprint of chrysanthemum buds.

The purpose of this study is to establish chromatographic fingerprints of chrysanthemum buds by CE-ED analysis. In this analysis, water and alcohol extraction methods will be successively employed to enhance extraction efficiency. Copper and carbon electrodes will be both used to guarantee that the fingerprints produced can encompass the main bioactive compounds. Two distinct chrysanthemum samples will be identified by the utility of the proposed fingerprint.

## 2. Materials and Methods

### 2.1. Materials and Reagents

Glucose and fructose were purchased from Sigma (St. Louis, MO, USA). Chlorogenic acid and luteolin were obtained from Shanghai Yuanye (Shanghai, China). Disodium tetraborate decahydrate (Na_2_B_4_O_7_·10H_2_O), H_3_BO_3_, phosphate salts, and sodium hydroxide (NaOH) were obtained from Shanghai Yuanye (Shanghai, China). All reagents were of analytical grade.

Glucose and fructose stock solutions were prepared in deionized water (Yancheng Chunyu Reagent Factory, Jiangsu, China). Chlorogenic acid and luteolin stock solutions were prepared with ethyl alcohol. The concentration of all stock solutions was 0.01 g mL^−1^. All analytes were diluted to the desired concentration in running buffer for CE analysis.

### 2.2. Sample Collection and Handling

Twelve batches of chrysanthemum bud samples were purchased from supermarkets in five main cultivation areas located in China (Table 1). The chrysanthemum buds were dried at room temperature and finely ground using a blender (Joyoung Limited by Share Ltd., Shandong, China). The analytes in chrysanthemum buds were extracted as follows.

First, the milled chrysanthemum buds (1 g) were suspended in 40 mL of deionized water and then ultrasonicated for 30 min to lyse the cells. Next, the mixture was heated at 90°C for 30 min to extract the water-soluble compounds. The suspension was cleared by centrifugation at 14800 rpm for 2 min using an Anke TGL-16C centrifuge (Shanghai Anting Instrument Factory, Shanghai, China), and the supernatant was filtered through a 0.22-*μ*m membrane to produce the polysaccharide fraction. To obtain the flavonoid fraction, the filtered residue was extracted with 50 mL 95% ethanol solution and ultrasonicated for 30 min. This suspension was centrifuged and stored as above. Before analysis, the samples were diluted with running buffer. All samples were prepared fresh every day.

### 2.3. Electrode Preparation

In this study, all employed electrodes were made in our laboratory.

A scrap copper wire (25 cm long, 0.3 mm diameter) was sealed into a soft glass capillary (10 cm long) with glue water. The capillary was cut perpendicular to its length to expose the wire at both ends. A copper electrode was used as soon as the glue solidified.

A lead inside a graphite pencil (4 cm long, 0.3 mm diameter) was first burnished and carefully wound with a polished copper wire. Then, the lead was sealed into a soft glass capillary with glue water. Finally, the capillary was cut perpendicular to its length to expose the lead and wire at each end of the capillary. The carbon electrode was used as soon as the glue solidified.

At the start of each experiment, both ends of the copper or carbon electrode were polished with extra fine carborundum paper followed by the sonication in deionized water using KQ-100KDE ultrasonic generator purchased from Kunshan Ultrasonic Instruments Co., Ltd. (Kunshan, China) before being placed in the cell.

### 2.4. CE-ED Instrument

CE analysis was performed on a laboratory-built CE-ED system [18]. A 30 kV high voltage power supply (Shanghai Institute of Nuclear Research, China) supplied the voltage between the ends of the capillary. The inlet end of the capillary was held at cathodic potential and the outlet end was maintained at ground. The inlet cell was filled with the separation running buffer, and the outlet end was placed in the detection cell filled with detection running buffer. A fused-silica capillary of 25 *μ*m (inner diameter) obtained from Hebei Yongnian Factory (Handan, China) was used for the separation. The samples were injected electrokinetically.

The design of the CE-ED system was based on the end-column approach. The working electrode (either copper or carbon) was placed at the outlet of the separation capillary, and detection was carried out in the reservoir containing the grounding electrode for the CE instrument. Before use, the surface of the working electrode was positioned carefully opposite to the capillary outlet using a micropositioner (Shanghai Lianyi Instrument Factory, China). A three-electrode cell system composed of the working electrode, a platinum auxiliary electrode, and a saturated calomel electrode (SCE) was employed along with a BAS LC-3D amperometric detector (Biochemical System, West Lafayette, IN, USA). The electropherograms were processed with the HW-2000 software (Shanghai Qianpu Microsoftware, China).

### 2.5. CE Analysis

As in previous CE-ED analyses [19], several key factors were investigated to find the optimal separation conditions. The running buffer was selected from Na_2_B_4_O_7_-H_3_BO_3_, phosphate salts, Na_2_B_4_O_7_-NaOH, and NaOH; pH and the concentration of the running buffer varied from 9 to 13 and from 10 to 50 mM, respectively; separation voltage ranged from 10 to 25 kV; the potential applied to copper working electrode ranged from 0.5 to 0.8 V, and the potential applied to carbon working electrode ranged from 0.8 to 1.1 V.

### 2.6. Data Analysis

The method was validated by identifying some key known compounds in chrysanthemum buds, such as chlorogenic acid, luteolin, glucose, and fructose. The relative standard deviations (RSDs), linearity, and detection limits of these compounds were calculated to determine the feasibility of this method.

Due to the novelty of fingerprinting analysis, only a few papers have been published on chemometrics [20]. In this study, data were analyzed with the professional software Computer-Aided Similarity Evaluation, which was developed based on chemometrics by the Research Center for the Modernization of Traditional Chinese Medicines (Central South University, Changsha, China). Ten batches of chrysanthemum buds were analyzed to establish the mean chromatograph as a representative standard fingerprint electropherogram. Data was analyzed using included angle cosine [21] and correlation coefficient [22] methods in order to compare their suitability for discriminating between chrysanthemum fingerprints.

The included angle cosine method considers the fingerprint spectrum data as a multidimensional space vector to convert the fingerprint spectrum similarity problem into the similarity between multidimensional vectors. The included angle cosine (cos⁡*θ*) is calculated by the following equation:

(1) $$\begin{array}{c} \cos\theta =\frac{\sum_{i=1}^{n}X_{i}Y_{i}}{\sqrt{\sum_{i=1}^{n}X_{i}^{2}}\sqrt{\sum_{i=1}^{n}Y_{i}^{2}}}, \end{array}$$

while the correlation coefficient (*γ*_1_), which measures the relationship between the two properties, is calculated by the following equation:

(2) $$\begin{array}{c} \gamma_{1}=\frac{\sum_{i=1}^{n}\left(X_{i}-\bar{X_{i}}\right)\left(Y_{i}-\bar{Y_{i}}\right)}{\sqrt{\sum_{i=1}^{n}{\left(X_{i}-\bar{X_{i}}\right)}^{2}\sum_{i=1}^{n}{\left(Y_{i}-\bar{Y_{i}}\right)}^{2}}}, \end{array}$$

where *X*_*i*_ and *Y*_*i*_ are the *i*th elements in the two different electropherograms (namely, *X* and *Y*, resp.) and *n* is the number of the elements in the electropherograms. $\bar{X}$ and $\bar{Y}$ are the mean values of the *n* elements in electropherograms *X* and *Y*, respectively.

### 2.7. Sample Identification

Under the optimum analysis conditions,* Chrysanthemum morifolium* and* Chrysanthemum indicum* obtained from local supermarkets were analyzed by CE-ED. The electropherograms were compared with the standard fingerprint of chrysanthemum buds to distinguish between various chrysanthemums.

## 3. Results and Discussion

### 3.1. CE Analysis

In order to achieve good separation of main components and quantify all of the bioactive chemical compounds in chrysanthemum buds, copper and carbon electrodes were utilized as the working electrode to analyze polysaccharides and flavonoids, respectively.

#### 3.1.1. Optimization Condition of CE with Carbon Working Electrode

The carbon electrode was used as the working electrode mainly to analyze flavonoid compounds in chrysanthemum bud samples. Running buffer selection was considered first because of its significant effect on separation. Na_2_B_4_O_7_-NaOH was chosen as the running buffer for its greater elution effect after comparing with the separation efficiency of Na_2_B_4_O_7_-H_3_BO_3_, phosphate salts, and Na_2_B_4_O_7_-NaOH.

The acidity and concentration of the running buffer also plays a key role in CE due to its effects on the zeta-potential (*ζ*), the electroosmotic flow (EOF), and the overall charge of the analytes, all of which impact the separation and migration time of the analytes. When the pH of the same running buffer in separation cell and detection cell was lower than 9.89, two standard compounds (chlorogenic acid and luteolin) could not be separated and there were few peaks, demonstrating poor separation efficiency. On the other hand, when the pH of the running buffer was higher than 12, the migration time was over 1 h. So pH 11.25 Na_2_B_4_O_7_-NaOH (including 3.1 × 10^−3^ g mL^−1^ boric acid ions) was selected as the optimum running buffer, balancing good separation with reasonable separation times.

The potential applied to the working electrode directly affected the sensitivity and detection limit of this method. Separation voltage affects the velocity of the electroosmotic flow and the migration time of the analytes. In the following analyses, the potential applied to the carbon electrode was maintained at 0.95 V, where the background current was not too high, while the signal-to-noise (S/N) ratio was the highest. Moreover, the working electrode demonstrated good stability and high reproducibility at this optimum potential.

The effect of the separation voltage on the migration time of the analytes was also studied. The results show that a higher separation voltage resulted in shorter migration times for all analytes but also resulted in increased baseline noise. In the following analyses, the separation voltage was maintained at 14 V.

#### 3.1.2. Optimization of CE with Copper Working Electrode

The copper electrode was used to analyze polysaccharide compounds in chrysanthemum buds. The optimal condition was selected with the same selection standards as for the carbon electrode. In order to obtain good separation and detection simultaneously [23], NaOH (pH 13.0) was selected as the optimal detection buffer because of the good response of the copper electrode in strong basic solution, and Na_2_B_4_O_7_ (pH 9.24, 7.63 × 10^−3^ g mL^−1^) was selected as the best separation buffer because it resulted in a good separation efficiency. The optimal potential to the copper working electrode was determined to be 0.67 V, and the optimal separation voltage was determined to be 20 kV.

### 3.2. Establishing the Fingerprint of Chrysanthemum Buds

**Figure 1:**
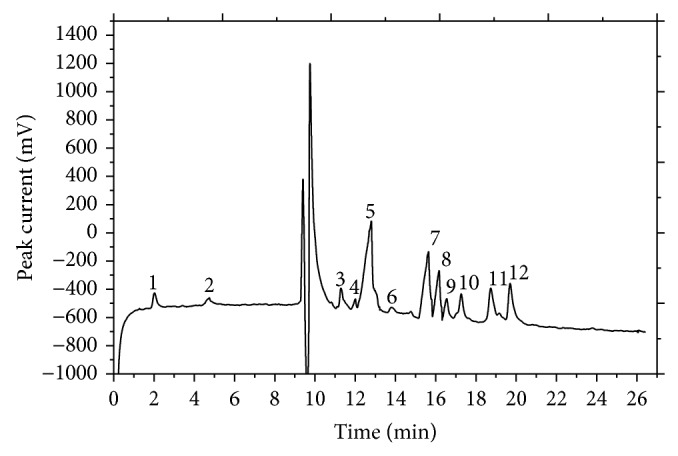
Standard fingerprint for chrysanthemum buds obtained from CE with the carbon working electrode. Peak 5: chlorogenic acid; Peak 6: luteolin. Working potential is 0.95 V (versus SCE); running buffer: Na_2_B_4_O_7_-NaOH (pH 11.25, 3.1 × 10^−3^ g mL^−1^ boric acid ions); separation voltage: 14 kV, inject time: 8 s.

**Figure 2:**
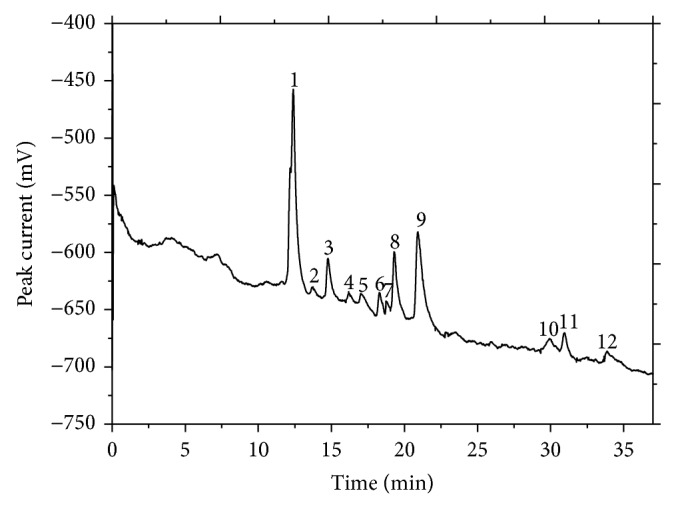
Standard fingerprint for chrysanthemum buds obtained from CE with the copper working electrode. Peak 8: glucose; Peak 11: fructose. Working potential: 0.67 V (versus SCE); separation buffer: Na_2_B_4_O_7_ (pH 9.24, 7.63 × 10^−3^ g mL^−1^); detection buffer: NaOH (pH 13.0); separation voltage: 20 kV, inject time: 8 s.

Because the similarity analysis determined that the 10 batches of chrysanthemum buds were highly similar, they were used to produce mean electropherograms for chrysanthemum buds using the copper working electrode (Figure 1) and the carbon working electrode (Figure 2). Together, these electropherograms comprise a comprehensive fingerprint of the bioactive compounds in chrysanthemum buds.

### 3.3. Identification of Markers and Method Validation

Some compounds found in chrysanthemum buds, such as chlorogenic acid, luteolin, glucose, and fructose, were selected as marker compounds to validate this technology. Peaks 8 and 11 in Figure 1 represent glucose and fructose, respectively. Peaks 5 and 6 in Figure 2 represent chlorogenic acid and luteolin, respectively. The reproducibility of the peak current was evaluated by repeatedly injecting a standard solution under the optimum conditions. The RSDs of the migration time were 1.83%, 0.57%, 2.13%, and 1.41%, respectively.

Additionally, a dilution series of standard solutions was also tested to measure the linearity of the current response for each of the four standard analytes. The linearity and detection limits are summarized in Table 2. As predicted, the observed reproducibility and detection limits of the four analytes were satisfactory.

### 3.4. Common Peaks Selection

Peaks found in all samples were assigned as “common peaks,” standing for the main characteristic compounds and representing the chemical profile of sample. According to the selection criterion of common peaks in Chinese herbs, common peaks were selected as those with a migration time RSD lower than 5% and a minimum relative peak area of less than 0.5%. Ten chrysanthemum bud samples were analyzed by CE with both the copper and carbon electrodes. 12 common peaks were chosen from each of the two CE electropherograms as the common peaks for chrysanthemum buds for a total of 24.

### 3.5. Similarity Analysis

The similarity of 10 batches of chrysanthemum bud samples from various locations was evaluated by correlation coefficient and the included angle cosine. The similarity between samples was calculated by the correlation coefficient method with the average of all samples as a standard, and the similarities between samples were calculated by the included angle cosine method with the average of all samples taken as a standard. If the value of angle cosine and correlation coefficient from samples are similar with *γ* > 0.90 and cos⁡*θ* > 0.95, the samples are assigned to the same origin.

From the data analysis in Tables 3 and 4, ten chrysanthemum bud samples (numbers 1–10) had high similarity even though the concentration of active compounds among samples was not at the same level. From the results it can be concluded that the ten samples belong to the same species even though they are obtained from different place and different years.

### 3.6. Application of Standardized Fingerprint for Identification

Fingerprinting analysis can be used to assess the quality of chrysanthemum buds that come from different sources. By examining the relative retention time and the relative peak area of the common peaks in a fingerprint, we can determine whether a raw herb is genuine. But the most important application of fingerprints is that they can be used to separate different chrysanthemum varieties from each other.

Under the optimal analysis conditions, two other chrysanthemum species (*Chrysanthemum morifolium* and* Chrysanthemum indicum*) were analyzed by this CE-ED method. By comparing their electropherograms with the standardized fingerprint of chrysanthemum buds (Tables 3 and 4), the distinctive features of each species have been identified. Some of the fingerprint common peaks are not found (carbon peaks 1, 2, 9, and 11; copper peaks 2, 5, 7, and 9) from both species. What is more, similarity analysis demonstrated that these species are significantly different from *γ* < 0.90 and cos⁡*θ* < 0.92, which are considered to be very different by the Chinese Pharmacopoeia Commission and in accordance with the actual varieties we bought from the supermarket.

The above-mentioned results indicate that this method is accurate, sensitive, and reproducible foridentification and quality assessment of chrysanthemum buds. Furthermore, these methods may be used in further research in other natural agricultural products.

## 4. Conclusion

In this study, an efficient fingerprinting of chrysanthemum buds was developed by CE coupled with double detection electrodes, which established a quality control protocol based on biochemical makeup for chrysanthemum buds. We hope that this study has provided an appropriate method not only to generate fingerprints of herbs, but also to identify and asses the quality of chrysanthemum buds.

## Figures and Tables

**Table 1:** Resource of ten chrysanthemum buds' sample.

Sample number	Name of sample	Source
1	King of chrysanthemum buds	Hangzhou, Zhejiang
2	Chrysanthemum buds	Huangshan, Anhui
3	Chrysanthemum buds	Sheyang, Jiangsu
4	Chrysanthemum buds	Tongxiang, Zhejiang
5	Chrysanthemum buds	Jiaozuo, Henan
6	Chrysanthemum buds	Linyi, Shandong
7	Chrysanthemum buds	Lin'an, Zhejiang
8	Chrysanthemum buds	Kunming, Yunnan
9	Chrysanthemum buds	Yulin, Guangxi
10	Chrysanthemum buds	Bozhou, Anhui
11	*Chrysanthemum morifolium *	Hangzhou, Zhejiang
12	*Chrysanthemum indicum *	Bozhou, Anhui

**Table 2:** Results of the regression analysis on the calibration curves and the detection limits.

Compound	Regression equation^1^	Correlation coefficient (*γ* ^2^)	Linear range (×10^−4^ g mL^−1^)	Detection^2^ limits (10^−7^ g mL^−1^)
Glucose	*y* = 4.1 × 10^9^ *x* + 3.882	0.997	0.02–2.0	3.5
Fructose	*y* = 1.2 × 10^9^ *x* − 1.127	0.998	0.02–2.0	1.8
Chlorogenic acid	*y* = 1.8 × 10^9^ *x* − 160.29	0.995	0.02–2.0	6.2
Luteolin	*y* = 6.0 × 10^9^ *x* + 9.3887	0.998	0.02–2.0	5.1

^1^ *y* is the peak area; *x* is the concentration of the analytes (g mL^−1^).

^2^The detection limits correspond to the concentrations of the signal-to-noise ratio of 3.

**Table 3:** Peak area of various samples analyzed by CE with carbon electrode.

Peak	Sample^1^											
	1#	2#	3#	4#	5#	6#	7#	8#	9#	10#	11#	12#
1	1412023	1645374	1663717	1458483	1546122	1354982	1841631	1567651	1103240	1534023	No found	No found
2	907835	851391	813486	921323	923131	921354	835468	947313	994135	903712	No found	No found
3	2149706	2216378	2484610	2462123	2237155	2148461	1975232	2567855	2657165	1956742	1944541	2383874
4	1171446	1942103	1431231	1321330	903493	1579132	913485	1683256	1134902	991324	1076725	959534
5	16923651	18792134	19715642	13544132	18713543	15463449	19738394	18974667	13598120	14792134	6937854	7194685
6	679752	721564	643485	624836	681324	647122	594321	646794	679123	708616	1441354	2273234
7	14524829	15461365	11763465	18741561	13675645	10764475	18467132	19467486	13268785	15679815	10645381	8269314
8	5137047	5464132	5164725	5844154	5132112	5487612	5616912	581361	512641	541341	4251672	4586133
9	2166917	1774315	1843242	2354987	2465135	1949314	1891654	1676312	2546138	1687105	No found	2156515
10	3685649	4013651	4164623	3348971	3254854	3845624	3945215	3521265	3842946	4015689	3498665	3372452
11	4633980	5163435	4831656	4137568	4513981	4861635	5013826	4254682	4821591	4879612	4157674	No found
12	5547956	5312486	5461358	5782923	5978613	5428913	5342381	5138198	6021567	6223365	4978784	5445845
*γ*	0.997	0.995	0.969	0.957	0.990	0.978	0.996	0.981	0.973	0.976	0.890	0.841
cos⁡*θ*	0.998	0.998	0.984	0.977	0.994	0.987	0.997	0.985	0.986	0.986	0.910	0.903

^1^Previous 10 batches (from number 1 to number 10) are real samples to establish the standard fingerprint; numbers 11 and 12 are the real samples to be assessed.

**Table 4:** Peak area of various samples analyzed by CE-copper electrode.

Peak	Sample											
	1#	2#	3#	4#	5#	6#	7#	8#	9#	10#	11#	12#
1	3747425	3624777	4358598	6876961	5709162	4521216	4452454	4021416	5601451	4335254	7511364	4679831
2	153880	85758	141249	105365	95080	98623	131639	108698	150123	106415	No found	65264
3	1080579	978309	655783	629236	896236	91636	970861	800012	609357	879412	1064891	501310
4	104520	75693	67969	95421	90413	102576	89563	101233	91975	84489	98699	58956
5	238491	147583	219547	187573	219576	167089	197456	129046	148643	177533	287789	No found
6	149385	237952	288587	189234	190643	289456	190121	188965	159758	189765	159137	251987
7	58153	73624	55581	74285	69210	57459	61361	57868	71698	69124	No found	No found
8	1478427	1881504	1675532	1086598	789865	979853	1376068	1678754	1586985	975097	2457040	1178621
9	2746590	2088749	1286953	2089796	2083654	1875916	2987542	2858155	2074764	3585875	No found	No found
10	268517	361587	291786	301765	369866	308716	345425	222752	357146	291578	368271	407981
11	406312	295709	454106	305136	416918	309572	451986	312476	281461	331653	334586	321786
12	454742	562069	356123	501685	542352	441875	501153	608754	409852	588743	541657	487819
*γ*	0.976	0.975	0.971	0.978	0.983	0.986	0.989	0.980	0.992	0.960	0.877	0.876
cos⁡*θ*	0.983	0.981	0.980	0.980	0.987	0.989	0.992	0.986	0.993	0.973	0.902	0.902

## References

[1] Liang X., Wu H., Su W. (2014). A rapid UPLC-PAD fingerprint analysis of chrysanthemum morifolium ramat combined with chemometrics methods. *Food Analytical Methods*.

[2] Zhang S., Dong S., Chi L., He P., Wang Q., Fang Y. (2008). Simultaneous determination of flavonoids in chrysanthemum by capillary zone electrophoresis with running buffer modifiers. *Talanta*.

[3] Lin L.-Z., Harnly J. M. (2010). Identification of the phenolic components of chrysanthemum flower (*Chrysanthemum morifolium* Ramat). *Food Chemistry*.

[4] Pharmacopoeia of People’s Republic of China (2010). *The State of Pharmacopoeia Commission of People’s Republic of China*.

[5] Dattajirao K. S., Narayana R. B., Babasaheb B. D. (1990). Chrysanthemum. *Postharvest Biotechnology of Flowers and Ornamental Plants*.

[6] Shunying Z., Yang Y., Huaidong Y., Yue Y., Guolin Z. (2005). Chemical composition and antimicrobial activity of the essential oils of *Chrysanthemum indicum*. *Journal of Ethnopharmacology*.

[7] CDER (2004). *Guidance for Industry Botanical Drug Products*.

[8] Commissioning Process Management Professional (CPMP) (2000). *Note for Guidance on Specifications: Test Procedures and Acceptance Criterion for Herbal Drugs, Herbal Drug Preparations, and Herbal Medicinal and Herbal Medicinal Products*.

[9] Drug Administration Bureau of China

[10] Ni Y., Zhang L., Churchill J., Kokot S. (2007). Application of high performance liquid chromatography for the profiling of complex chemical mixtures with the aid of chemometrics. *Talanta*.

[11] Hajimahmoodi M., Heyden Y. V., Sadeghi N., Jannat B., Oveisi M. R., Shahbazian S. (2005). Gas-chromatographic fatty-acid fingerprints and partial least squares modeling as a basis for the simultaneous determination of edible oil mixtures. *Talanta*.

[12] Gu M., Su Z., Ouyang F. (2006). Fingerprinting of Salvia miltiorrhiza Bunge by thin-layer chromatography scan compared with high speed countercurrent chromatography. *Journal of Liquid Chromatography and Related Technologies*.

[13] Liang X., Wu H., Su W. (2014). A rapid UPLC-PAD fingerprint analysis of *Chrysanthemum morifolium* Ramat combined with chemometrics methods. *Food Analytical Methods*.

[14] Gu M., Zhang S., Su Z., Chen Y., Ouyang F. (2004). Fingerprinting of Salvia miltiorrhiza Bunge by non-aqueous capillary electrophoresis compared with high-speed counter-current chromatography. *Journal of Chromatography A*.

[15] Schmid M. G. (2012). Chiral metal-ion complexes for enantioseparation by capillary electrophoresis and capillary electrochromatography: a selective review. *Journal of Chromatography A*.

[16] Deeb S. E., Wätzig H., El-Hady D. A. (2013). Capillary electrophoresis to investigate biopharmaceuticals and pharmaceutically-relevant binding properties. *TrAC—Trends in Analytical Chemistry*.

[17] Chen G., Zhu Y., Wang Y., Xu X., Lu T. (2006). Determination of bioactive constituents in traditional Chinese medicines by CE with electrochemical detection. *Current Medicinal Chemistry*.

[18] Ye J., Baldwin R. P. (1993). Amperometric detection in capillary electrophoresis with normal size electrodes. *Analytical Chemistry*.

[19] Xing X., Shi X., Zhang H., Wang W., Ye J. (2009). Determination of diethylene glycol in toothpaste by capillary electrophoresis with electrochemical detection. *Microchimica Acta*.

[20] Goodarzi M., Russell P. J., Heyden Y. V. (2013). Similarity analyses of chromatographic herbal fingerprints: a review. *Analytica Chimica Acta*.

[21] Yang B. J., Chen J. H., Lee F. S.-C., Wang X. (2008). GC-MS fingerprints for discrimination of *Ligusticum chuanxiong* from Angelica. *Journal of Separation Science*.

[22] Jin Y., Liang T., Fu Q. (2009). Fingerprint analysis of Ligusticum chuanxiong using hydrophilic interaction chromatography and reversed-phase liquid chromatography. *Journal of Chromatography A*.

[23] Xing X., Cao Y. (2007). Determination of 3-chloro-1,2-propanediol in soy sauces by capillary electrophoresis with electrochemical detection. *Food Control*.

